# P23 Optimised Patient centred Team based Interdisciplinary Management Approach to UTI—OPTIMA-UTI: an antimicrobial management team and infection prevention and control team collaborative

**DOI:** 10.1093/jacamr/dlae217.027

**Published:** 2025-01-23

**Authors:** Jo McEwen, David Shepherd, Mhairi Steven, Heather Kennedy, Pamela Davidson, Alison Davie, Benjamin Parcell, Busi Mooka, Sophie Fox, Olivia Davidson, Charis Marwick

**Affiliations:** NHS Tayside, Dundee, Scotland; NHS Tayside, Dundee, Scotland; NHS Tayside, Dundee, Scotland; NHS Tayside, Dundee, Scotland; NHS Tayside, Dundee, Scotland; NHS Tayside, Dundee, Scotland; NHS Tayside, Dundee, Scotland; NHS Tayside, Dundee, Scotland; NHS Tayside, Dundee, Scotland; NHS Tayside, Dundee, Scotland; NHS Tayside, Dundee, Scotland; University of Dundee, Dundee, Scotland

## Abstract

**Background:**

Catheter associated urinary tract infection (CAUTI) and urinary tract infection (UTI) are leading causes of *Escherichia coli* bacteraemia (ECB) within Scotland, resulting in increased risk of adverse outcomes, cost, increased exposure to antibiotics and a decrease in quality of care. NHS Tayside have higher rates of urinary catheterization and ECB than other Scottish Health Boards. OPTIMA-UTI is a collaborative quality improvement initiative aimed at reducing the burden of hospital associated (HAI) UTI and ECB. Improving choice of catheter product and compliance with NHS Tayside CAUTI bundle were two areas of initial focus. The choice between long-term (LTC) and short-term catheters (STC) is critical in clinical practice, influencing patient outcomes, infection rates, and healthcare costs. CAUTI bundles not only document care delivery but are also evidence-based tools universally recommended for the prevention of CAUTI.

**Objectives:**

The aim of the overall OPTIMA-UTI programme is to improve patient outcomes, UTI/CAUTI diagnosis and management. The objectives of the work presented are: (i) improve catheter product selection; and (ii) improve CAUTI bundle compliance.

**Methods:**

Four acute pilot areas (medical, surgical and two frailty/medicine for the elderly units), forming the main routes of admission to other inpatient areas, were selected for inclusion. Outcome and process measures were captured across the baseline phase (March 24 – June 24), intervention phase (June 24 – November 24) and observation phase (November 24 – January 25). The intervention included the following. *Education & training:* Evidence based resources, developed for ward-based staff education and training targeting system ‘point of care’ elements were delivered collaboratively by the Senior Antimicrobial Stewardship Nurse and the Infection Prevention and Control Nurse. *Environmental changes:* Catheter storage areas were redesigned to facilitate correct catheter selection at point of insertion guiding staff around the appropriateness of LTC versus STC selection. CAUTI bundles were relocated next to catheters for ease of access at point of insertion. ECB rates attributable to the pilot areas and overall acute HAI ECBs were monitored as a core clinical outcome for the overall OPTIMA-UTI project. Process outcomes were fed back in education sessions for clinical staff.

**Results:**

There appears to be a reduction in the proportion of LTCs and a rise in the proportion of STCs, particularly towards the end of the intervention phase as practice embeds. However, overall numbers of catheters have risen. Improvements were noted to the percentage of patients having a CAUTI bundle in place during the intervention phase. No ECBs were attributed to any of the pilot sites since the intervention phase and the number of HAI ECBs across all acute wards is lower after the intervention, although this is only a short timeframe.

**Conclusions:**

Whilst improvements were noted around catheter selection, further work is required to reduce the overall prevalence and burden of catheters within the organization which is likely to require multi-professional engagement. Learning and resources from OPTIMA-UTI have been adopted as part of a Board-wide ECB improvement plan.

